# IKKα is involved in kidney recovery and regeneration of acute ischemia/reperfusion injury in mice through IL10-producing regulatory T cells

**DOI:** 10.1242/dmm.018200

**Published:** 2015-07-01

**Authors:** Xin Wan, Li-Jun Hou, Li-Yuan Zhang, Wen-Juan Huang, Lin Liu, Qian Zhang, Bo Hu, Wen Chen, Xin Chen, Chang-Chun Cao

**Affiliations:** 1 Division of Nephrology, Department of Medicine, Nanjing First Hospital, Nanjing Medical University, Nanjing 210006, China; 2 Division of Neurosurgery, Department of Surgery andShanghai Neurosurgical Institute, The Second Military Medical University, Changzheng Hospital, Shanghai 200003, China; 3 Division of Nephrology, Department of Medicine, Affiliated Lianyungang Clinical Medical College of Nanjing Medical University, Lianyungang 222002, China; 4 Division of Cardiovascular Surgery, Department of Surgery, Nanjing First Hospital, Nanjing Medical University, Nanjing 210006, China

**Keywords:** IκB kinase α, Kidney, Ischemia-reperfusion injury, Repair, IL10, T regular cell, Indoleamine 2, 3-dioxygenase

## Abstract

The recovery phase after kidney ischemia/reperfusion (IR) injury is often associated with the suppression of inflammation and the proliferation of tubular epithelial cells (TECs). The duration of this phase is often determined by the suppression of inflammation and the proliferation of TECs. Several lines of evidence suggest that IκB kinase α (IKKα) not only promotes the production of anti-inflammatory factors and/or prevents the production of inflammatory factors, but also induces the accompanying cell differentiation and regeneration, and suppresses inflammation. We therefore hypothesized that IKKα could participate in the kidney repair after IR injury and have used a mouse model of acute kidney injury (AKI) to test this. We found that IKKα mediated the repair of the kidney via infiltrated regulatory T (Treg) cells, which can produce anti-inflammatory cytokine IL10, and that IKKα also increased the proliferation of the surviving TECs and suppressed of inflammation. In addition, the expression of indoleamine 2,3-dioxygenase (IDO) in TECs is consistent with the infiltration of IL10-producing Treg cells. We conclude that IKKα is involved in kidney recovery and regeneration through the Treg cells that can produce IL10, which might be a potential therapeutic target that can be used to promote kidney repair after IR injury.

## Abstract (precis)

**Summary:** IKKα in the kidney recovery and regeneration via regulatory T cells secreting IL10.

## INTRODUCTION

It is well known that ischemia-reperfusion (IR) injury can cause acute kidney injury (AKI) (Bonventre and Weinberg, 2003). The renal tubules are very sensitive to acute ischemia and a prolonged ischemic episode leads to epithelial cell death in proximal parts of renal tubules. Despite this, the kidney is still able to recover to its normal tissue function and structure after prolonged ischemia (El Sabbahy and Vaidya, 2011; Kulkarni et al., 2014). After the inflammation that follows IR injury, the impaired kidney often heals (Benigni et al., 2010; El Sabbahy and Vaidya, 2011). In fact, proinflammatory/anti-inflammatory and damage/repair processes are closely intertwined during the entire recovery process (Bonventre and Zuk, 2004; Duffield et al., 2005; Benigni et al., 2010; El Sabbahy and Vaidya, 2011; Kulkarni et al., 2014).

Several possibilities have been suggested to explain the origin of the regenerating epithelial cells, such as surviving tubular epithelial cells (TECs), bone marrow stem cells and a population of renal mesenchymal stem cells (Duffield et al., 2005). Increasing evidence suggests that the surviving TECs play an active role in the response to inflammation, contributing to the repair of the epithelium (Bonventre and Weinberg, 2003; Duffield et al., 2005).

The repair process in kidney after IR injury is characterized by two major events: the resolution of local inflammation and the restoration of cell numbers in response to the surviving TECs undergoing dedifferentiation and migrating along the basement membrane. Their final re-differentiation into mature tubular cells restores functional integrity. Thus, it is clear that a reduction in inflammation and replenishment of the tubular epithelium are both needed for repair; this reduction and replenishment is triggered by the same signaling cascades during the repair phase (Humphreys et al., 2008; Benigni et al., 2010; Bonventre and Yang, 2011).

The IκB kinase (IKK) complex constitutes two catalytic subunits: IKKα and IKKβ. It regulates activation of the transcriptional factor NFκB, which plays a crucial role in inflammation (Baldwin, 1996; Ghosh and Karin, 2002). Evidence shows that IKKβ mediates activation of NFκB in response to pro-inflammatory stimuli via the canonical pathway, and that the activation of NFκB plays a central role in the induction of the expression of pro-inflammatory cytokine genes that contribute to tissue injury during reperfusion (Baldwin, 1996; Ghosh and Karin, 2002). We and others have observed that inhibition of NFκB or silencing of IKKβ in an experimental model of renal IR injury markedly reduced tubule lesions and monocyte/macrophage infiltration (Moss et al., 2007; Wan et al., 2011). However, Lawrence et al. found that the effects of NFκB were greatly dependent on the time course of inflammation (Lawrence et al., 2001). Inhibiting NFκB during the resolution phase of pleural inflammation was shown to exert severe adverse effects (Lawrence et al., 2005). Thus, NFκB is involved in both the initiation of inflammation and the resolution process. In this process, IKKα promotes the regression of acute inflammation through an alternative NFκB activation pathway (Lawrence et al., 2001, 2005). In addition, IKKα can manipulate a number of genes involved in transformation, angiogenesis, and proliferation and differentiation of keratinocytes and tumor endothelial cells by a mechanism independent of its kinase activity and of NFκB activity (Hu et al., 2001; Luo et al., 2007; DeBusk et al., 2008; Descargues et al., 2008).

**TRANSLATIONAL IMPACT**

**Clinical issue**

Ischemia-reperfusion (IR) injury is an important cause of acute kidney injury, which is a common condition, especially in older people. Although ischemia leads to extensive epithelial cell death in the proximal tubule, after IR injury and subsequent inflammation, the impaired kidney often heals. The repair process is accompanied by two major events: the resolution of local inflammation and the restoration of the functional integrity of the kidney. This latter process involves the dedifferentiation and proliferation of the surviving tubular epithelial cells (TECs), followed by their re-differentiation. TECs therefore represent a target for therapies designed to promote the recovery of renal function.

**Results**

Here, the authors use a mouse model of IR injury to investigate the role of IKKα in kidney repair after IR injury. The IκB kinase (IKK) complex contains two catalytic subunits (IKKα and IKKβ) and regulates the activation of the transcriptional factor NFκB, which plays a crucial role in inflammation. Several lines of evidence suggest that IKKα not only promotes the production of anti-inflammatory factors and/or prevents the production of inflammatory factors, but also induces cell differentiation and regeneration. The authors report that IKKα, which is distributed abundantly along the proximal tubule, is essential for kidney repair. They show that IKKα mediates the infiltration of Treg cells (a subtype of lymphocytes that maintains homeostasis in the immune system) into the kidney, and that Treg cells are the major source of the anti-inflammatory cytokine IL10, increased levels of which are associated with kidney recovery. Finally, they show IKKα is required for the proliferation of surviving TECs.

**Implications and future directions**

These results provide important new insights into the involvement of IKKα in kidney recovery and regeneration. By showing that IKKα mediates kidney repair and regeneration by inducing the infiltration of Treg cells and the subsequent production of the anti-inflammatory cytokine IL10, these findings identify potential therapeutic targets that might help to promote kidney repair after IR injury and provide novel clues for both preventing and treating IR injury.

Indoleamine 2,3-dioxygenase (IDO) is a tryptophan-metabolizing enzyme with diverse physiological functions, including modulation of regulatory T (Treg) cells (Tas et al., 2007). The noncanonical NFκB signaling is essential for expression of IDO (Tas et al., 2007). Recently, a crucial role for IDO was demonstrated in the protection of cardiac allografts and in the prolongation of allergenic corneal graft survival (Chen et al., 2012; Bock et al., 2013). The synergistic crosslinked interplay between IDO and Treg cells is essential for these events to occur (Sucher et al., 2012).

CD4^+^CD25^+^Foxp3^+^ Treg cells, a subtype of T lymphocytes that predominantly produce IL10 and TGFβ, are crucial for the maintenance of homeostasis in the immune system. These lymphocytes are also responsible for the suppression of the adaptive immune response and are implicated in pathology of autoimmune diseases such as crescentic GN and adriamycin-induced nephropathy (Wolf et al., 2005; Tas et al., 2007; Costantino et al., 2008; Bettini and Vignali, 2009; Notley and Ehrenstein, 2010; Chen et al., 2012; Sucher et al., 2012; Bock et al., 2013). Recent evidence shows that Treg cells can suppress innate immunity in renal IR injury by inhibiting adriamycin-induced macrophage accumulation (Mahajan et al., 2006; Lee et al., 2010). However, the mechanisms by which Treg cells could inhibit the suppression of inflammation in renal IR injury and in the subsequent repair process are largely unknown. The anti-inflammatory cytokine IL10, which is released by Treg cells, B cells, dendritic cells and TECs, may be a key participant in protecting against IR injury due to its immunosuppressive effect (Deng et al., 2001; Bamboat et al., 2010; Ostmann et al., 2013).

We hypothesized that the activation of IKKα-dependent NFκB noncanonical pathway could drive the resolution of inflammation, as well as subsequent tissue regeneration or repair during the recovery phase of AKI. In this paper, we have identified unknown anti-inflammatory and pro-regeneratory properties of the IKKα pathway underlying TEC regeneration in AKI.

## RESULTS

### IKKα-dependent NFκB noncanonical pathway is activated in the recovery phase of kidney IR injury

**Fig. 1.**
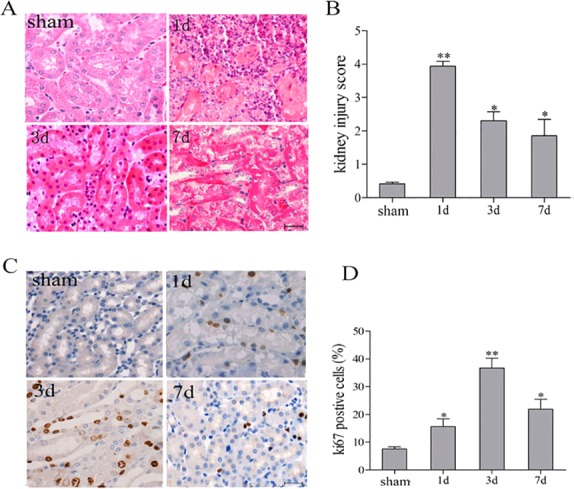
**Renal histological changes, proliferation and renal function following renal IR injury.** C57BL/6 mice underwent unilateral renal pedicle clamping for 45 min, followed by reperfusion. (A,C) Conventional histological analysis of Hematoxylin and Eosin (A) and the tubular proliferative indicator Ki67 (C) stained kidney sections was performed on days 1, 3 and 7 following IR injury. (B,D) Histological damage (B) and Ki67 levels (D) were scored in a blind manner. Data are presented as mean±s.d. (*n*=6 mice). **P<0.01, *P<0.05 versus sham treated.

The NFκB signaling pathway plays a crucial role in post-ischemic events that take place in the kidney. Most of the focus has been placed on the role of IKKβ in canonical NFκB pathway, which triggers an innate immunity response that determines the extent of damage at the early phase of AKI (Ghosh and Karin, 2002; Wan et al., 2011). To test a putative role of IKKα on inflammation resolution and epithelial repair, we used a model of acute IR-induced kidney injury, in which the mice underwent unilateral renal pedicle clamping. Renal tubules in the outer medulla are particularly sensitive to ischemia and are susceptible to severe damage and death. In this model, we evaluated the time course of the kidney repair processes at 1, 3 and 7 days after IR injury. We selected these particular time points due to the rapid progression of repair pathology. The pathological changes are at their worst by the first day, whereas repair is evident by the third day after IR injury (Fig. 1A). TEC proliferation reached its peak on day 3, declined by day 7 (Fig. 1C) and nearly returned to baseline levels by day 14 (data not shown). Thus, we evaluated the mice on day 3 after IR injury, when repair was observable but still incomplete.

We evaluated kidneys for tubular pathology, TEC regeneration and renal function in this disease model and in sham-treated mice. On day 1 after IR injury, kidneys showed signs of severe tubular pathology (dilated tubules, casts) in the outer medulla and cortex when compared with sham-treated mice (Fig. 1A). More evident infiltration of inflammatory cells existed on day 1 and gradually reduced until day 3. The nuclear division in the TECs was widespread. On day 7, the infiltration of inflammatory cells and nuclear division were decreased, indicating the decline in the severity of kidney injury. Because the renal pedicle clamping was unilateral (instead of bilateral) in this animal model, changes in two prominent systemic indicators, serum creatinine (Scr) and blood urea nitrogen (BUN), were within the normal range (data not shown).

**Fig. 2.**
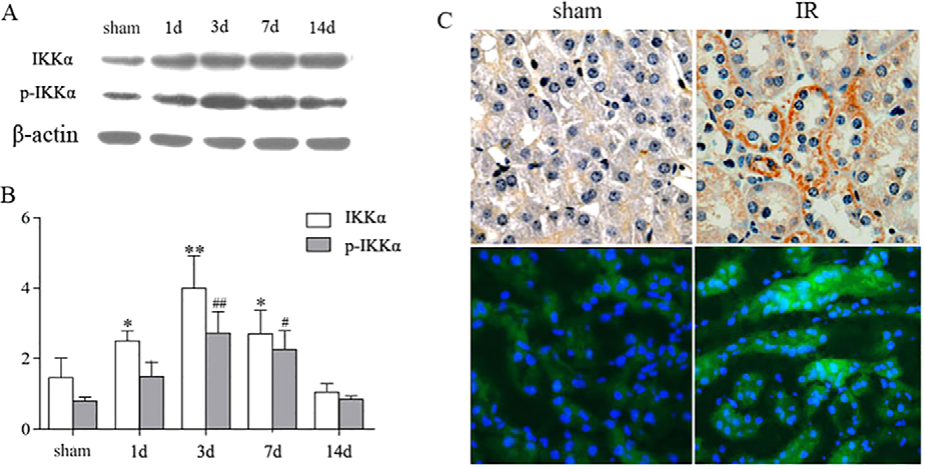
**The expression of IKKα on TECs in the repair phase after renal IR injury.** C57BL/6 mice underwent unilateral renal pedicle clamping for 45 min, followed by reperfusion. The kidneys were harvested on days 1, 3, 7 and 14, respectively. (A) The expression of IKKα and p-IKKα was analyzed by western blotting. (B) Levels of IKKα in the kidneys after normalization to β-actin. (C) Expression of IKKα by immunohistochemistry (top) and immunofluorescence staining (bottom). Representative images of IKKα staining are distributed in the renal TECs and tubule interstitium. Data are presented as mean±s.d. (*n*=6 mice). **P<0.01, *P<0.05 versus sham treated for IKKα/β-actin; and ^##^*P*<0.01, ^#^*P*<0.05 versus sham treated for p-IKKα/β-actin.

To investigate the potential effects of IKKα on the renal repair mechanism after IR injury, the expression of both total and phosphorylated IKKα (p-IKKα) were analyzed by western blotting. Minimal levels of IKKα were detected in the sham-treated kidney. By contrast, markedly increased IKKα protein expression was noted in the sample from day 1 to day 7 after kidney recovery, with a peak on day 3 and a gradual decline towards the baseline (Fig. 2A).

Moreover, we localized IKKα by detecting its expression using immunohistochemistry and immunofluorescence staining. The positive staining along the proximal tubule demonstrated its abundant expression in this region (Fig. 2C), which was consistent with the western blotting results.

**Fig. 3.**
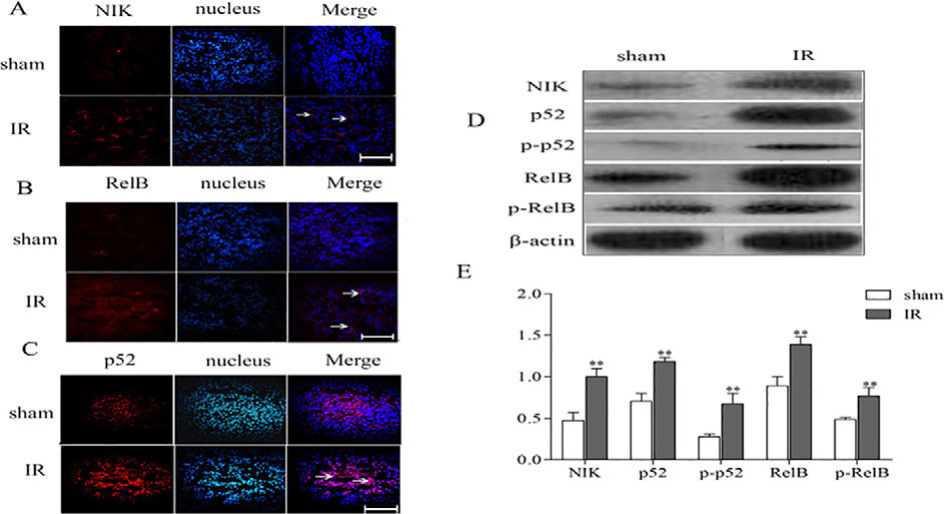
**The expressions of NFκB cascade components in the repair phase of renal IR injury.** C57BL/6 mice underwent unilateral renal pedicle clamping for 45 min, followed by reperfusion. (A-C) The kidneys were harvested on day 3. Immunofluorescence staining for NIK (A), p52 (B) and RelB (C) was performed. Positive staining for NIK and RelB was distributed in the renal TECs and tubulointerstitium; the positive staining for p52 was located in the nucleus. The sham-treated kidney exhibited faint staining for these cytokines, whereas IR caused the stronger staining on day 3. Scale bars: 20 μm. Arrows indicate positive results. (D) Protein levels of NIK, p52, p-p52, RelB and p-RelB were measured by western blotting. (E) Expression levels of NIK, p52, p-p52, RelB and p-RelB in the kidney, after normalization to β-actin were all significantly decreased in the IR kidneys compared with sham-treated kidneys. Data are presented as mean±s.d. (*n*=6 mice). **P<0.01 versus the sham treated.

The IKKα noncanonical pathway starts with phosphorylation of IKKα dimers, which in turn leads to phosphorylation of p100, cleavage of p100 into p52, translocation of p52/RelB complexes to the nucleus and finally the activation of NFκB-inducible kinase (NIK). Consistent with the expression of IKKα, the expression of NIK, p52, p-p52, RelB and p-RelB was also detected in surviving TECs in repair phase by immunofluorescence or western blotting (Fig. 3).

### Silencing IKKα impairs inflammation resolution and TEC proliferation

**Fig. 4.**
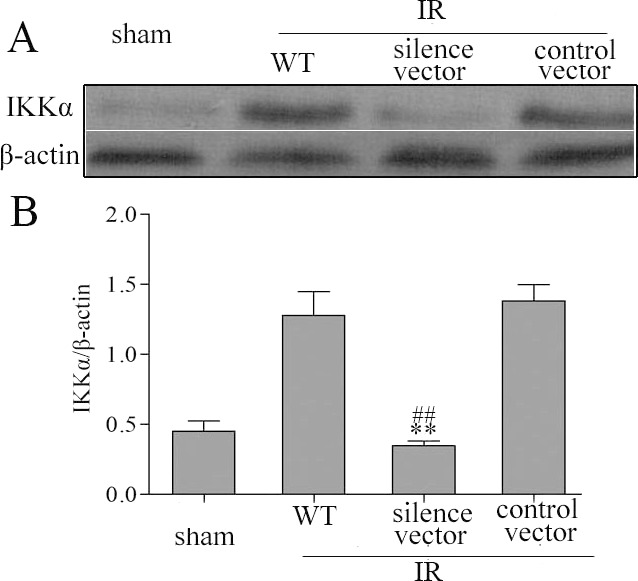
**IKKα expression was effectively suppressed by IKKα-shRNA *in vivo* and *in vitro.*** (A) C57BL/6 mice underwent: a sham-treated operation; IR produced by unilateral renal pedicle clamping for 45 min followed by reperfusion (WT); or IR conducted 2 weeks after renal parenchyma injection of GV118-GFP-shRNA-IKKα lentiviral vectors (silence vector) or GV118-GFP-lentiviral vector (control vector). The kidneys were harvested on day 3. (B) NRK52E cells were cultured with lymphotoxin-LIGHT to activate the IKKα pathway (lane 1), or transfected with control vectors (lane 2) or GV118-GFP-shRNA-IKKα lentiviral vectors (lane 3). IKKα protein levels in the kidneys were detected by western blotting after normalization to β-actin levels. Data are expressed as mean±s.d. (*n*=6 mice), **P<0.01 versus wild type, ^##^*P*<0.01 versus control vector.

**Fig. 5.**
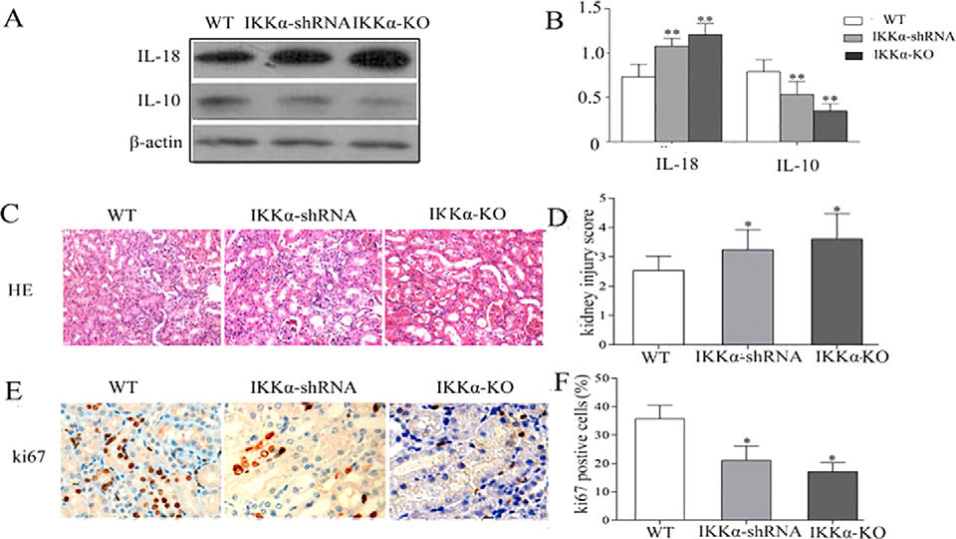
**Silencing IKKα impairs inflammation resolution and tubular proliferation of IR kidneys.** C57BL/6 wild-type mice underwent IR by unilateral renal pedicle clamping for 45 min followed by reperfusion (WT) or IR conducted 2 weeks after renal parenchyma injection of GV118-GFP-shRNA-IKKα lentiviral vectors (IKKα-shRNA). We also carried out unilateral ischemic injury in IKKα-null mice (IKKα-KO, IKKα^fl/fl^, Cre^+/−^ mice). The kidneys were harvested on day 3. (A,B) Protein levels of IL10 and IL18 were measured by western blotting. (C) Hematoxylin and Eosin staining of renal cortex. Kidneys from IKKα-shRNA or IKKα-KO mice showed significantly more tubular damage than wild-type kidneys. (D) The histological damage score was determined in a blind manner. (E) The cells stained reddish-brown represent Ki67-positive cells, reflecting the proliferation process; (F) they were quantified in a blind manner. Data are presented as mean±s.d. (*n*=6 mice). *P<0.05 versus wild type, **P<0.01 versus wild type.

Inflammation resolution and TEC proliferation are two hallmarks of kidney recovery after IR injury. To further investigate the role of IKKα in the kidney repair process, we silenced IKKα by intrarenal delivery of GV118-GFP-IKKα shRNA or GV118-GFP-lentiviral vector. The vector was used as a control 2 weeks before IR, and cohorts of mice were killed on day 3. To investigate whether intrarenal delivery of IKKα-shRNA could downregulate IKKα expression after IR injury, we harvested the kidneys for protein isolation. Expression of IKKα was dramatically reduced after GV118-GFP-IKKα shRNA intrarenal delivery, compared with wild-type mice after IR injury (Fig. 4A). Similarly, the expression of IKKα was significantly suppressed after transfection with GV118-GFP-IKKα shRNA *in vitro* (Fig. 4B). No significant effect on IKKβ was observed when IKKα was either silenced *in vitro* or knocked down *in vivo* (supplementary material Fig. S1). In this way, silencing IKKα significantly delayed inflammation resolution and TEC regeneration. It was illustrated by increased levels of IL18 (Fig. 5A), decreased levels of IL10 (Fig. 5A), the higher index of kidney injury score (Fig. 5D) and the lower number of proliferation marker Ki-67cells (Fig. 5E). Furthermore, we induced unilateral IR in IKKα-null (genotype IKKα^fl/fl^, Cre^+/−^) mice. These mice also displayed delayed inflammation regression and impaired TEC regeneration accompanied by higher IL18 levels on day 3 (Fig. 5B). Together, these data support the theory that IKKα mediates renal repair by promoting inflammation regression and enhancing TEC regeneration in recovery phase following IR injury.

### Increased IL10 and Treg cells are associated with kidney recovery

**Fig. 6.**
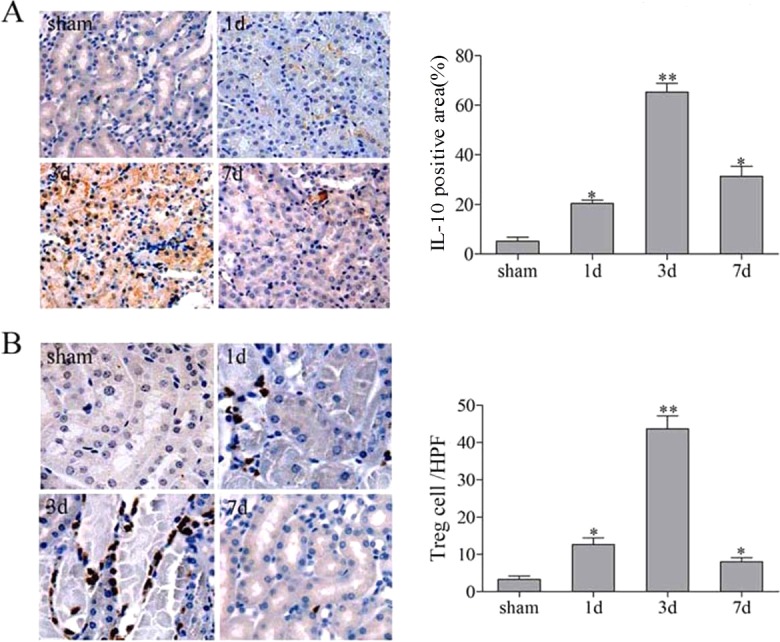
**The expression of IL10 and Foxp3^+^ in the repair phase of renal IR injury.** C57BL/6 mice underwent a sham-treated operation or unilateral renal pedicle clamping for 45 min, followed by reperfusion. The kidney tissues were harvested on days 1, 3 and 7. Representative immunehistochemistry staining of IL10 and Foxp3^+^ in the kidney from sham-treated or IR groups. (A) IL10 was located predominantly inside kidney vascular structures and in the interstitium, and was markedly increased on day 3. (B) The staining for Foxp3 was located in the nucleus with a peak on day 3. Data are presented as mean±s.d. (*n*=6 mice). *P<0.05 versus sham treated; **P<0.01 versus sham treated.

The ability of Treg cells to traffic to the areas of inflammation and their ability to suppress innate immunity in kidney IR injury has been demonstrated previously (Kinsey et al., 2009). We hypothesized that kidney IR injury would also cause accumulation of Treg cells in the kidney. Thus, the number of Treg cells was examined by immunohistochemistry. Data showed that the accumulation of Foxp3^+^ Treg cells started as early as 24 h after reperfusion and that they remained throughout the whole healing phase. Levels were markedly increased on day 3 of reperfusion compared with any other time points in the repair phase. The accumulation occurred mainly in the tubule interstitial nuclei (Fig. 6B).

Growing evidence indicates that IL10 protects the kidney from IR injury by invoking both anti-inflammatory and proliferative responses (Bamboat et al., 2010). IL10 expression was detected in the post-ischemic kidney at 24 h, with a peak at day 3 following reperfusion. On day 7, it was still higher and could be detected until day 14 (data not shown) during the repair process. Notably, it was located predominantly inside kidney vascular structures and in the interstitium (Fig. 6A).

### Treg cells are the major source of IL10 following kidney IR injury

The above data indicate that IL10 and Treg cells might mediate kidney repair by promoting inflammation regression and TEC proliferation. We next investigated whether Treg cells are the major source of IL10 following kidney IR injury.

**Fig. 7.**
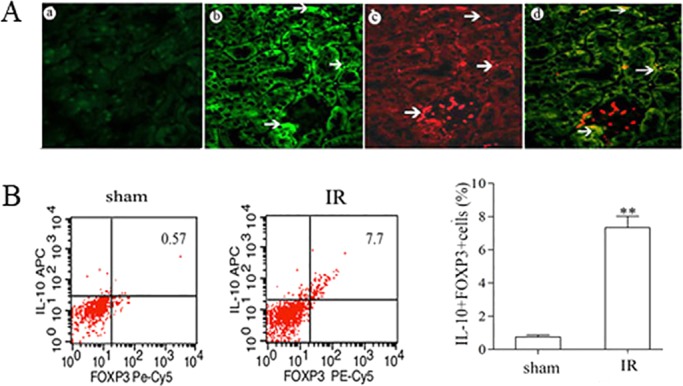
**Foxp3^+^ cells are the source of IL10 in the recovery phase after renal IR injury.** C57BL/6 mice underwent IR by unilateral renal pedicle clamping for 45 min, followed by reperfusion. (A) On day 3, the kidneys were harvested for immunofluorescence staining and flow cytometry analysis. Dual immunofluorescence staining for IL10 (b; green, arrows indicate TECs and infiltrated cells) and Foxp3 (c; red, arrows indicate infiltrated cell nuclei), indicating that the majority of IL10-positive cells were Foxp3-positive Treg cells (d). (a) No antibody control. (B) Representative flow cytometry results for Foxp3 and IL10. The percentage of Foxp3- and IL10-positive cells were increased in IR kidneys after 3 days compared with sham-treated kidneys. Data are presented as mean±s.d. (*n*=6 mice). **P<0.01 versus sham treated.

In addition to the pronounced expression of IL10 in TECs, a substantial amount of IL10 was seen in infiltrated cells in the interstitium. Immunofluorescence staining and flow cytometry were used to validate the sources of IL10. IL10 immunoreactivity was localized to the immunocytes identified by their positive staining for the Treg cell marker Foxp3^+^ using dual immunofluorescence (Fig. 7B) and also confirmed by flow cytometry (Fig. 7A), suggesting that IL10 was partly produced by the Treg cells.

**Fig. 8.**
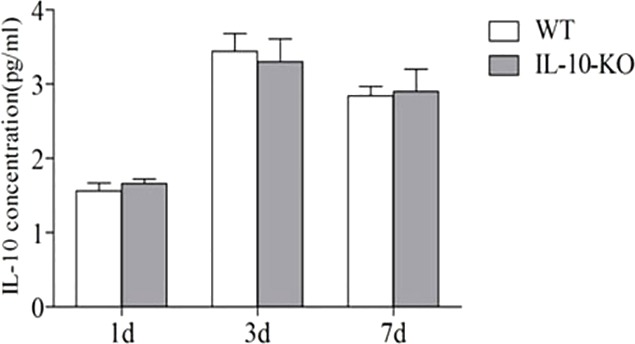
**Adoptive transfer of Treg cells rescues IL10 production in *Il10*^−/−^ mice after renal IR injury.** C57BL/6 mice underwent IR by unilateral renal pedicle clamping for 45 min, followed by reperfusion (WT). Treg cells were isolated and purified from spleens of C57BL/6 mice, and then injected intravenously into *Il10*^−/−^ mice (IL-10-KO) at 18 h before IR induction. The level of IL10 in *Il10*^−/−^ was comparable with the kidneys in WT-IR on days 1, 3 and 7. Data are presented as mean±s.d. (*n*=6 mice). There was no significant difference in IL10 levels between wild type and *Il10*^−/−^ mice.

To further delineate whether the effects of IL10 were derived from the Treg cells, we used a transfer strategy. Treg cells from spleens of wild-type mice were isolated, purified and injected intravenously to *Il10*^−/−^ mice at 18 h before IR induction. IL10 levels in the kidneys of IR injury mice were assessed by ELISA. On day 1, increased IL10 levels were observed (particularly at day 3 and day 7), which was comparable with the results of the kidneys after IR injury in wild-type mice (Fig. 8). These findings suggest that infiltrated Treg cells contribute to AKI repair during the recovery phase by secreting IL10.

### IKKα is essential for the infiltration of Treg cells in the kidney recovery phase

**Fig. 9.**
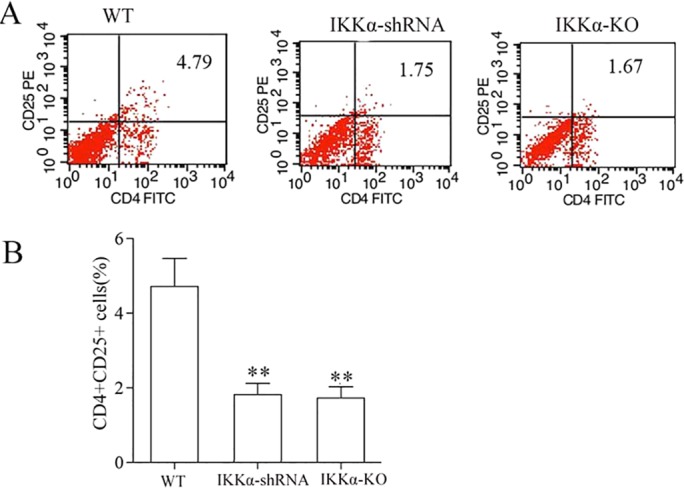
**Percentage of kidney-infiltrating Treg cells after renal IR.** Total kidney cells were gated on the CD4^+^ and CD25^+^ population before the analysis shown. (A) The percentage of CD4^+^CD25^+^ Treg cells was decreased in IKKα-shRNA or IKKα-KO (IKKα^fl/fl^, Cre^+/−^) kidneys compared with wild type after 3 days, as assessed by flow cytometry analysis. (B) The percentage of CD4^+^CD25^+^ Treg cells in kidneys was determined. Data are presented as mean±s.d. (*n*=6 mice). **P<0.01 versus wild type.

To further emphasize the importance of IKKα expression in TECs for infiltration of Treg cells in the kidney recovery phase, we induced IR injury in IKKα-null (genotype IKKα^fl/fl^, Cre^+/−^) mice and then compared kidney pathologies. Our results showed that, on day 1, both groups displayed serious injuries, and no significant difference was noted between the wild-type and IKKα-null mice (supplementary material Fig. S2). However, on day 3 after reperfusion, there was less recovery from IR injury in the mutant mice, as indicated by still higher acute tubular necrosis (ATN) scores, when compared with wild-type mice (Fig. 5D). Immunohistochemistry showed a greater accumulation of Treg cells on day 3 after reperfusion in wild-type mice, predominantly inside kidney vascular structures and in the interstitium of the cortex and outer medulla (Fig. 6B). Quantitative analysis of Treg cells was then tested by flow cytometry. The results showed the population of Treg cells to be ∼0.76%±0.21% in sham-treated kidneys and 4.79%±0.52% in IR-treated wild-type mice, whereas Treg cells accounted for 1.75%±0.46 and 1.67%±0.41% of the population in IR-treated IKKα-shRNA mice and IKKα-null mice, respectively (Fig. 9). In addition, the number of infiltrated Treg cells was significantly decreased in IKKα-null mice on day 3 following reperfusion.

### IKKα regulated the expression of IDO in TECS *in vitro* and *in vivo*

IDO is a tryptophan-metabolizing enzyme, the expression of which requires noncanonical NFκB signaling and has several physiological activities, such as suppressing activated T cells. Recently, it has been demonstrated that IDO plays a crucial role in cardiac allograft protection, increasing the number of Treg cells and prolonging allogeneic corneal graft survival. Therefore, we hypothesized that the expression of IDO in the kidney recovery phase is regulated by the IKKα noncanonical pathway and promotes kidney recovery by modulating Treg cell effector function and self-propagation.

**Fig. 10.**
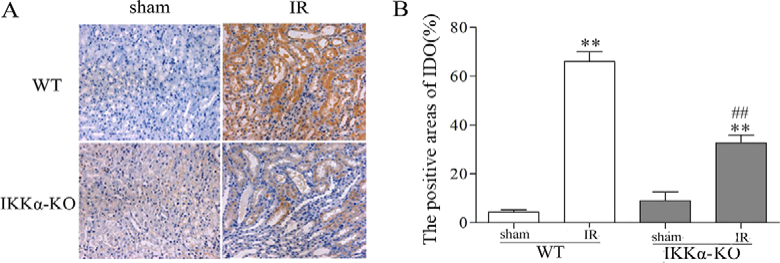
**IKKα regulates the expression of IDO in kidneys after renal IR injury.** C57BL/6 (wild type) or IKKα-null mice (IKKα-KO, IKKα^fl/fl^, Cre^+/−^ mice) underwent sham-treated operation or ischemia-reperfusion by unilateral renal pedicle clamping for 45 min, followed by reperfusion (IR). The kidney tissues were harvested on day 3. (A) Representative images of IDO infiltrated the interstitium and renal TECs of IR kidneys. Kidneys of wild-type mice had intense and diffuse IDO staining within the tubule interstitium. However, enhancement of IDO expression is mediated by IKKα, as evidenced by minimum expression of IDO in IKKα-KO mice. (B) The IDO-positive areas were determined in a blind manner. Data are presented as mean±s.d. (*n*=6 mice). **P<0.01 versus respective sham treated; ^##^*P*<0.01 versus WT-IR.

Immunohistochemical assessment indicated that IDO was weakly expressed within TEC of sham-treated kidneys. By contrast, kidneys on day 3 after IR injury had intense and diffuse IDO staining within the tubule interstitium. But lower expression of IDO occurred in IKKα-null mice (Fig. 10) and in the wild-type mice treated with intrarenal delivery of LV-GFP-IKKα shRNA (which can silence IKKα) 2 weeks before pedicle clamping (data not shown).

**Fig. 11.**
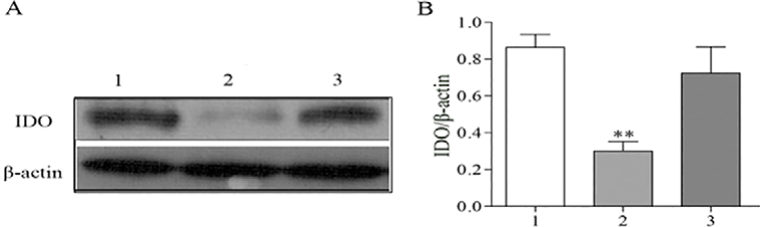
**IKKα regulates the expression of IDO *in vitro*.** NRK52E cells were cultured with lymphotoxin-LIGHT to active the IKKα pathway (lane 1), transfected with GV118-GFP-shRNA-IKKα lentiviral vectors (lane 2) or transfected with control vectors (lane 3). (A) The protein levels of IDO were measured by western blotting. (B) The levels of IDO were normalized to β-actin. Data are presented as mean±s.d., **P<0.01 versus the control vector.

To further determine the effect of IKKα on IDO expression in cultured TECs, which is stimulated by the noncanonical pathway agonist homologous lymphotoxin-LIGHT, we silenced IKKα using GV118-GFP-shRNA-IKKα lentiviral vectors. As shown in Fig. 11, the *in vitro* experiment demonstrated an inhibition of IDO expression in IKKα knocked-down cells. All these results suggest that IKKα upregulates IDO expression during the repair phase.

## DISCUSSION

Inflammation resolution and TEC proliferation are two hallmarks in the kidney recovery phase following IR injury (Bonventre and Weinberg, 2003; Bonventre and Zuk, 2004; Duffield et al., 2005; Benigni et al., 2010; El Sabbahy and Vaidya, 2011; Kulkarni et al., 2014). At the initial phase, IR injury involves a sterile inflammatory response that somewhat contributes to the tubular cell damage. Subsequently, damaged necrotic tubular cells act as the predominant trigger for amplification of the inflammatory response by damage-associated molecular patterns (DAMPs), which initiate the influx of various inflammatory cells into the kidney (Kulkarni et al., 2014). In general, pro-/anti-inflammatory and damage/repair processes are closely intertwined in response to renal IR injury (Bonventre and Zuk, 2004; Duffield et al., 2005; Benigni et al., 2010). In the late recovery phase, the resolution of sterile inflammation and repair from acute tubular injury involves recruitment of immune cells, such as intratubular progenitor cells or bone marrow-derived stem cells, which may act as important sources of pro-regeneratory and growth factors (Bonventre and Zuk, 2004; Duffield et al., 2005; Humphreys et al., 2008; Benigni et al., 2010). There is increasing evidence that surviving TECs play an important role in the repair in response to AKI. Indeed, within a few hours of IR injury, surviving TECs enter the cell cycle and initiate the dedifferentiation, migration and proliferation that eventually leads to kidney recovery (Humphreys et al., 2008).

To allow tissue recovery in sterile tissue injuries, a number of counter regulatory mechanisms exist to prevent the immune reaction from becoming pathologically excessive. These mechanisms include limiting the activation of surviving TECs and infiltrated immune cells (Humphreys et al., 2008; Kulkarni et al., 2014). As an important signaling pathway, the activation of NFκB participates in the initiation and termination of inflammation, and subsequent tissue regeneration or repair process. The contribution of classical activation of NFκB in kidney injury during reperfusion has drawn much attention (Wan et al., 2011; Kundumani-Sridharan et al., 2012; Kulkarni et al., 2014). However, the detailed actions of IKK have yet to be explained.

Using a unilateral IR injury model, we found that proliferation and differentiation of surviving TECs begins 24 h after kidney IR injury. Furthermore, our findings demonstrate that both the inflammatory resolution and epithelial tubule proliferation during the repair stage were greatly dependent on IKKα-dependent noncanonical NFκB pathway. Interestingly, we also found that the major components in the NFκB noncanonical pathway, such as IKKα, NIK, p52, p-p52, RelB and p-RelB, were also expressed exclusively in surviving TECs during the repair phase. Silencing or knocking down IKKα in TECs worsened histological damage and delayed tubular regeneration on day 3 after IR injury. It prompted the activation of other tubular epithelial pathways involved in the repair of renal ischemic injury. Both the early and late phases of kidney IR injury are characterized by infiltration of T lymphocytes, which, like macrophages and DCs, can facilitate injury but also promote repair after IR injury (Gandolfo et al., 2009; Li et al., 2012).

Treg cells were initially identified as CD4^+^CD25^Hi^ lymphocytes and are now defined by the expression of Foxp3 on the surface of this subset of cells (Fontenot et al., 2003). These lymphocytes have anti-inflammatory abilities and an intrinsic protective function in renal IR injury (Ostmann et al., 2013). They are trafficked to and accumulate in the area of inflammation in the kidney as a consequence of the IR injury, which in turn should protect the kidney from subsequent damage and facilitate kidney repair in the recovery phase (Gandolfo et al., 2009; Bonventre and Yang, 2011). These actions can be managed by the release of anti-inflammatory cytokines such as IL10, by the modulation of pro-inflammatory cytokine production of other T cell subsets or by the production of extracellular adenosine (Gandolfo et al., 2009; Kinsey et al., 2009; Ostmann et al., 2013).

In this experiment, using flow cytometry technology, we observed that kidney Treg cell numbers were significantly infiltrated 3 days after IR injury. This result is in agreement with recent findings of Gandolfo et al., who reported that there was a significant trafficking of Treg cells into the kidneys after 3 and 10 days in the model of ischemic acute kidney injury (Gandolfo et al., 2009).

In addition, double immunofluorescence staining was used to test whether IL10 and Treg cells were colocalized. Consistently, the result showed a high expression of IL10 on Treg cells. To further confirm that IL10 was derived from the Treg cells, we used a transfer strategy. As expected, increased kidney IR injury was observed in *Il10*^−/−^ mice, as measured by BUN levels and ATN. It has previously been found that adoptive transfer of wild-type Treg cells into *Il10*^−/−^ mice could alleviate kidney IR injury (Kinsey et al., 2009). By contrast, after adoptive transfer of *Il10*^−/−^ Treg cells, the extent of damage did not change, implying an important role for IL10 from Treg cells in the recovery from IR injury. Together, these results demonstrate that Treg cells function to promote the kidney repair in response to the inflammation and dysfunction associated with IR injury, likely through the production of IL10. Although IL10 is produced by Treg cells, ite is also produced by damaged renal tubular cells and by other T cells and macrophages. Indeed, a weak expression of IL10 in surviving TECs was also observed in our study.

Despite suffering from passive injury, the TECs are involved in the inflammatory response to IR injury in the kidney (Humphreys et al., 2008; Kulkarni et al., 2014). In addition to producing pro-inflammatory and chemotactic cytokines, which activate inflammatory cells, surviving TECs were associated not only with activation of inflammatory cells through production of pro-inflammatory and chemotactic cytokines, but also with regulation of T lymphocyte activity through expression of Toll-like receptors (TLRs), complement and its receptors, and co-stimulatory molecules (Humphreys et al., 2008; Bonventre and Yang, 2011; Kulkarni et al., 2014). We have found that the decreased level of major components in the NFκB noncanonical cascade was consistent with the trend observed in Treg cells and IL10 in kidney after silencing IKKα or in IKKα-null mice. It is suggested that, in the recovery phase, activation of the noncanonical NFκB pathway in surviving TECs could actually suppress inflammation and promote regeneration by Treg cell infiltration that favors IL10 production.

Recently, studies have demonstrated that the noncanonical NFκB pathway induces the IDO-mediated expression of DCs by differentiated Treg cells, acting as an activator of the noncanonical NFkB pathway (Puccetti and Grohmann, 2007; Manches et al., 2012). In fact, IDO is mainly expressed in the DCs, Treg cells, macrophages, epithelial cells and endothelial cells.

In this study, we first show that the expression of IDO is proportional to the number of infiltrated Treg cells in the recovery phase following kidney IR injury. We reveal that surviving TECs are the source of IDO production, which might be beneficial to kidney repair and inflammation regression in the repair phase after IR injury. It is interesting to find a discrepancy between our findings and those of and Mohib and Jevnikar (Mohib et al., 2008). The latter found that IDO expression augments kidney injury following renal IR. The discrepancy may come from the different time points chosen in these two studies. As suggested by Masoumy et al., IDO expression alters depending on the timing of ischemia and on the micro environmental conditions (Masoumy et al., 2014). In Mohib and Jevnikar's study, they observed the expression of IDO in renal tissue from 2 h to24 h, the early stage of kidney IR injury. At this stage, the proinflammatory response is the major pathological process. By contrast, we observed that upregulation of IDO existed 3 days after reperfusion, which is in the later phase or repair stage of IR injury. At this late phase, the anti-inflammatory response is the predominant reaction in the body. We speculate that the dual functions of IDO were consistent with immunocytes in the processes of kidney IR.

In models of cardiac allograft transplantation, intragraft Treg cells have been shown to cluster around IDO-expressing endothelial cells (Thebault et al., 2007; Sucher et al., 2012), indicating an intimate cell-cell interaction among Treg cells and other type of cells. In the present study, which was limited by the detection sensitivity of immunohistochemical analysis, the expression of IDO was increased as the number of infiltrating cells increased. Whether Treg cells were the IDO-producing cells need further investigation.

Further analysis of the mechanism underlying the induction and accumulation of the IDO-producing cells during the recovery phase is currently under investigation. During the last stage, we detected the expression of IDO in TECs by silencing IKKα *in vitro* and *in vivo*. We proved that IDO expression in TECs is inhibited after silencing IKKα, which is consistent with the recovery and the decrease in the number of IL10-producing Treg cells.

### Conclusion

We conclude that IKKα-dependent noncanonical NFκB pathway activation has the potential to drive the resolution of inflammation as well as subsequent TEC repair during the recovery phase of AKI through secretion of IL10 from Treg cells.

## MATERIALS AND METHODS

### Animals

Homozygous IKKα-floxed mice (C57BL/6 background) were obtained from the Jackson Laboratories. Transgenic mice that expressed Cre recombinase contain a kidney-specific Ksp-IKKα promoter (ksp-Cre). By mating IKKα-floxed mice with Ksp-Cre transgenic mice, conditional knockout mice (Ksp-IKKα^−/−^) in which the IKKα gene was specifically disrupted in renal TECs (genotype IKKα^fl/fl^, Cre^+/−^) were created. *Il10* gene knockout (*Il10*^−/−^) mice were originally obtained from Jackson Laboratories and bred in the Nanjing Medical University Experimental Animal Center. Sex- and age-matched C57BL/6 wild-type mice (aged 6-8 weeks old and weighing 20-25 g) were bred as controls. Animals received humane care according to guidelines set by the Institutional Animal Care and Use Committee of the Nanjing Medical University.

### Surgical procedures used for the renal ischemic model

The study protocols were approved by the Institutional Animal Care and Use Committee of the Nanjing Medical University. Mice were anesthetized with an intraperitoneal injection of chloral hydrate (10%, 0.35 ml/10 g). After abdominal midline incision, the left renal pedicle was bluntly dissected and clamped with a micro vascular clamp for 45 min. During the procedure, mice were kept well hydrated with warm sterile saline at the constant temperature (37°C). After clamps were removed, the wounds were sutured and the mice were allowed to recover with free access to food and water. Sham-operated mice underwent the identical procedure, except that clamping of the renal pedicles was omitted. Cohorts of mice were killed on days 1, 3, 7 and 14 after surgery. The post-ischemic kidneys and sham-treated kidneys were harvested and stored at −80°C until needed for further analysis. For silencing of IKKα, mice were divided into four groups (*n*=6 each): (1) ischemia-reperfusion and recovery (IR); (2) IR treated with a GFP-IKKα-encoding lentivirus (silence vector); (3) IR treated with GFP-encoding lentivirus (control vector); and (4) sham-treated group, in which the left renal pedicle was bluntly dissected without clamping. The GV118-GFP-shRNA-IKKα lentiviral vectors were purchased (GeneChem) and the target shRNA sequences were as follows: IKKα, 5′-ggaauaaauacagguucuctt-3′ (forward); 5′-gagaaccuguauuuauucctg-3′ (reverse). A GV118-GFP-lentiviral vector was used as a control. *In vivo* virus transduction to interference IKKα in C57BL/6 mice was performed as described elsewhere (Nakamura et al., 2004). In anesthetized mice, after temporary occlusion of the left renal pedicle, a 31 G needle was inserted at the lower pole of the left kidney parallel to the long axis and was carefully pushed toward the upper pole. After the needle was slowly removed, 50 µl filter-purified lentivirus cocktail (GFP or GFP-IKKα, 1×10^5^ IU/µl) was injected. Mice were subjected to renal IR 2 weeks after virus injection, as described above.

### Assessment of kidney damage

Outer medullary tubular necrosis was assessed using Hematoxylin and Eosin staining as described elsewhere (Day et al., 2006; Kinsey et al., 2009). Briefly, stained tissue sections were scored using a previously described semi-quantitative scale designed to blindly evaluate the degree of tubular necrosis. Acute tubular necrosis (ATN) scores were defined as tubular necrosis, tubular dilatation and/or atrophy, inflammatory cell infiltrate or cellular edema (Cao et al., 2004; Day et al., 2006; Humphreys et al., 2008), and graded on a scale of 0 to 4. The degree of damage was judged as follows: 0, normal kidney; 1, minimal necrosis (<5% involvement of the cortex or outer medulla); 2, mild necrosis (5-25% involvement of the cortex or medulla); 3, moderate necrosis (25-75% involvement of the cortex or medulla); and 4, severe necrosis (>75% involvement of the cortex or medulla).

### Immunohistochemical staining

Immunohistochemistry staining was performed on formalin-fixed kidney tissues. Sections were deparaffinized with xylene and rehydrated in a graded alcohol series and then placed in PBS (pH 7.5). The microwave antigen retrieval procedure (citrate buffer, pH 6.0) was performed for 10 min. After that, sections were immersed in 3% hydrogen peroxide for 10 min to block endogenous peroxidase, then treated with 3% BSA (diluted in PBS) for blocking nonspeciﬁc binding sites, and incubated overnight at 4°C with the following primary antibodies: rabbit anti-mouse ki67 (1:2000, Abcam), IKKα (1:200, Abcam), NIK (1:200, Santa Cruz Biotechnology), p52 (1:200, Santa Cruz Biotechnology), RelB (1:300, Cell Signaling), IL10 (1:500, Abbiotec) and Foxp3^+^ (1:100, Santa Cruz Biotechnology). The next day, these slides were incubated with horseradish peroxidase (HRP)-conjugated anti-rabbit or anti-rat secondary antibody for 1 h at room temperature. 3,3-Diaminobenzidine tetrahydrochloride was applied to the slides for developing brown color. Counterstaining was carried out with Eosin. All slides contained duplicate sections, from which one served as a control for secondary antibody binding speciﬁcity. The positive areas were measured in five randomly chosen fields, and quantified blindly using an Olympus BX-URA2 camera.

### Immunofluorescence staining

Sequential frozen sections (4 μm) were treated with Triton X-100 (1:1000, diluted in PBS) on ice for 10 min to enhance antigen permeability, and then blocked with 3% BSA to decrease nonspecific staining. Sections were incubated overnight at 4°C with affinity-purified polyclonal rabbit anti-IKKα antibody (1:100, diluted in PBS, Santa Cruz Biotechnology), polyclonal rabbit anti-IL10 antibody (1:200, diluted in PBS, Abbiotec), polyclonal rabbit anti-NIK antibody (1:200, diluted in PBS, Santa Cruz Biotechnology), polyclonal rabbit anti-p52 antibody (1:200, diluted in PBS, Santa Cruz Biotechnology), polyclonal rabbit anti-RelB antibody (1:200, diluted in PBS, Cell Signaling) and polyclonal rabbit anti-Foxp3 antibody (1:100, diluted in PBS, Santa Cruz Biotechnology). The secondary antibody used was a FITC-conjugated anti-rabbit antibody. Slides were also incubated with Hoechst Dye solution (Invitrogen, Carlsbad) for 5 s in the dark for further counterstaining (Stokman et al., 2005). Finally, slides were analyzed and evaluated by the average of staining intensity in 400× magnification on fluorescence microscopic examination by Olympus BX-URA2.

### Flow cytometry and adoptive transfer

The isolation of leukocytes from murine kidneys was performed as described elsewhere (Kinsey et al., 2009) with slight modifications. In brief, kidneys were finely minced, suspended with PBS (pH 7.2) and then passed through a 200 mm nylon mesh. Single-cell suspensions were separated using Percoll density gradient centrifugation (2000 ***g*** for 30 min). The leukocyte-enriched cell suspension was aspirated from the Percoll interface, washed twice with PBS and centrifuged at 2000 ***g*** for 5 min. CD4+ CD25+ Treg cells were stained, fixed and permeabilized using the eBioscience Foxp3+ buffer according to the manufacturer's protocol (eBioscience). Intracellular cytokine staining was performed for IL10. Kidneys were harvested and finely minced for leukocyte isolation as described above. Extracellular staining for CD4 and CD25 was performed based on the manufacturer's protocol. Then cells were fixed and permeabilized using the BD Cytofix/perm kit according to the manufacturer's protocol and stained with mouse IL10 APC (BD Biosciences).

In adoptive transfer experiments, splenic CD4^+^CD25^+^ Treg cells were isolated from wild-type mice using magnetic bead separation (CD4^+^CD25^+^ regulatory T cell kit; Stemcell Technologies). The mean purity of CD4^+^CD25^+^ Treg cells was 90%, as measured with flow cytometry. Approximately 1.2×10^6^ cells were injected intravenously into *Il10*^−/−^ mice via the tail vein 18 h before induction of IR injury as described (Day et al., 2006; Madge et al., 2008). The mice were sacrificed on days 1, 3 and 7.

### Western blotting analysis

The total proteins were extracted from kidney tissues as described elsewhere (Ostmann et al., 2013). Protein concentrations were measured using a BCA protein assay kit (Keygen). Equal amounts (50 μg) of lysate proteins were separated on 8% gel (for detection of IKKα and p-IKKα, respectively), transferred onto PVDF membrane, blocked with 5% nonfat dry milk in TBST buffer (TBS, pH 7.5, 0.1% Tween 20) for 1 h and then incubated overnight at 4°C with rabbit polyclonal anti-IKKα antibody (1:300, diluted in TBST, Santa Cruz Biotechnology), rabbit polyclonal anti-p-IKKα antibody (1:200, diluted in TBST, Santa Cruz Biotechnology), anti-p52 (1:500, Santa Cruz Biotechnology), anti-p-p52 (1:150, Santa Cruz Biotechnology), anti-RelB (1:500, Cell Signaling); anti-p-RelB (1:300, Cell Signaling), anti-NIK (1:500) from Santa Cruz Biotechnology or rabbit polyclonal anti-β-actin antibody (1:500, diluted in TBST, BIOS). On the following day, after extensive washing with TBST buffer, the membranes were incubated with HRP-conjugated anti-rabbit secondary antibody (1:5000, diluted in TBST, KeyGEN Biotechnology) for 2 h, then developed with the use of an enhanced chemiluminescence system (ECL kit, KeyGEN Biotechnology) and captured on light-sensitive Kodak imaging film.

### Cell culture and silencing IKKα *in vitro*

NRK52E cells purchased from American Type Culture Collection (Rockville) and grown in Dulbecco's modified Eagle's medium (DMEM) (Hyclone) supplemented with 10% heat-inactivated fetal bovine serum (FBS) (Gibco BRL) and 50 U/ml penicillin (Gibco BRL). Cells were then selected for successful transfection by GV118-GFP-shRNA-IKKα lentiviral vectors and treated with the homologous lymphotoxin-LIGHT (R&D Systems). GV118-GFP-lentiviral vectors were used as a control. Viral supernatant was harvested with 1 μg/ml polybrene and stored at −80°C. Cells were incubated with viral supernatant overnight and replaced with standard growth medium during the day. This infection protocol was repeated through six rounds, with cell passage as required. Whole-cell lysates were then prepared and immunoblotted for IKKα expression as described elsewhere (Madge et al., 2008).

### Enzyme-linked immunosorbent assay

Kidney tissues were obtained from mice for each treatment groups on days 1, 3, and 7 after mice IR induction (see above), respectively. Enzyme-linked immunosorbent assays (ELISAs) was performed to measure the concentrations of IL10 using Quantikine immunoassay kits (Uscn Life Science). Equal amounts of kidney tissues were homogenized with equal amounts of PBS (w/v). After that, the homogenates were centrifugated for 5 min at 5000 ***g*** and the supernatant removed and assayed immediately as described in the manufacturer's protocol.

### Statistical analysis

Data are expressed as mean±s.d. as appropriate. Statistical analyses were performed using ANOVA test, followed by the Student-Neumann-Keuls test. The differences were evaluated with SPSS 16.0 software (SPSS). Two-sided *P*<0.05 was considered statistically significant.

## Supplementary Material

Supplementary Material
