# Supplementary material for: IKKα is involved in kidney recovery and regeneration of acute ischemia/reperfusion injury in mice through IL10-producing regulatory T cells
Source: Dis Model Mech. 2015 Jul 1;8(7):733–42. doi: 10.1242/dmm.018200 (PMC4486855; doi:10.1242/dmm.018200)
Supplement: Supplementary Material [file supp_8_7_733__index.html]

Supplementary Material 

# IKKα is involved in kidney recovery and regeneration of acute ischemia/reperfusion injury in mice through IL10-producing regulatory T cells

## DMM018200 Supplementary Material

- Supplementary Material
