# Supplementary material for: IKKα is involved in kidney recovery and regeneration of acute ischemia/reperfusion injury in mice through IL10-producing regulatory T cells
Source: Dis Model Mech. 2015 Jul 1;8(7):733–42. doi: 10.1242/dmm.018200 (PMC4486855; doi:10.1242/dmm.018200)
Supplement: Supplementary Material [file supp_018200_DMM018200supp.pdf]

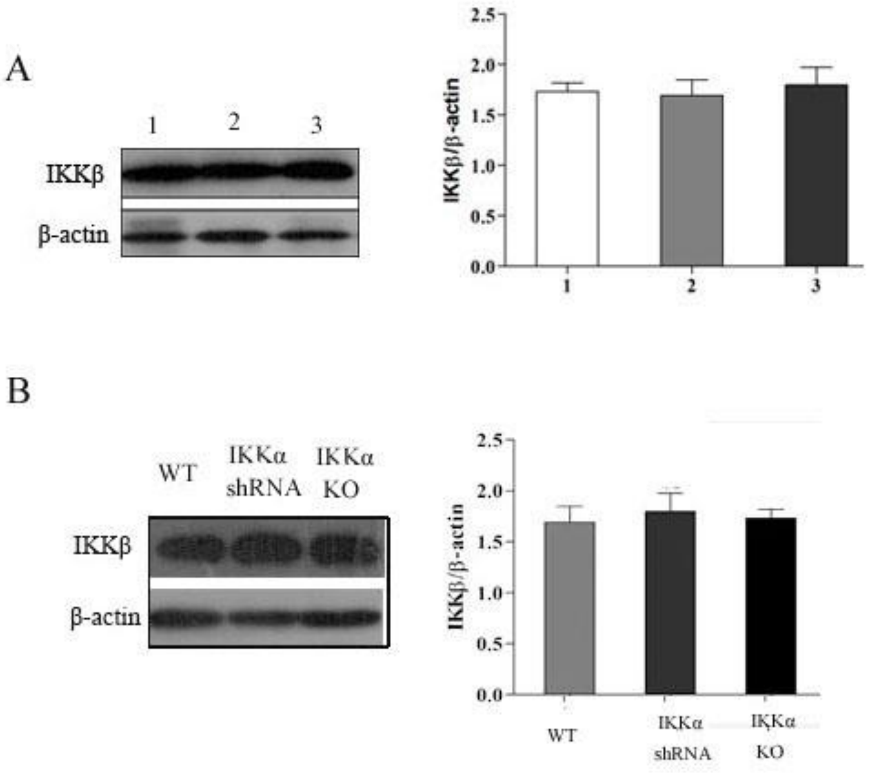

Suppl Fig 1. The expression of IKKβ was invariant in the IKKα silenced cells or knocked-down mice.

A: NRK52E cells were cultured with lymphotoxin-LIGHT to active IKKα pathway (lane 1), or transfected with either control vectors (lane 2) or GV118-GFP-shRNA-IKKα lentiviral vectors (lane 3). B: C57BL/6 wild-type mice underwent IR by unilateral renal pedicle clamping for 5 min, followed by reperfusion (WT), or conducted IR 2 weeks after renal parenchyma injection of GV118-GFP-shRNA-IKKα lentiviral vectors (IKKα-shRNA). Unilateral ischemic injury was also performed in IKKα$^{-/-}$ mice (IKKα-KO). The kidneys were harvested on day 3. The expression levels of IKKβ protein were measured by western blotting, and were normalized to β-actin.

Data are presented as mean ± SD. No significant difference was found among groups.

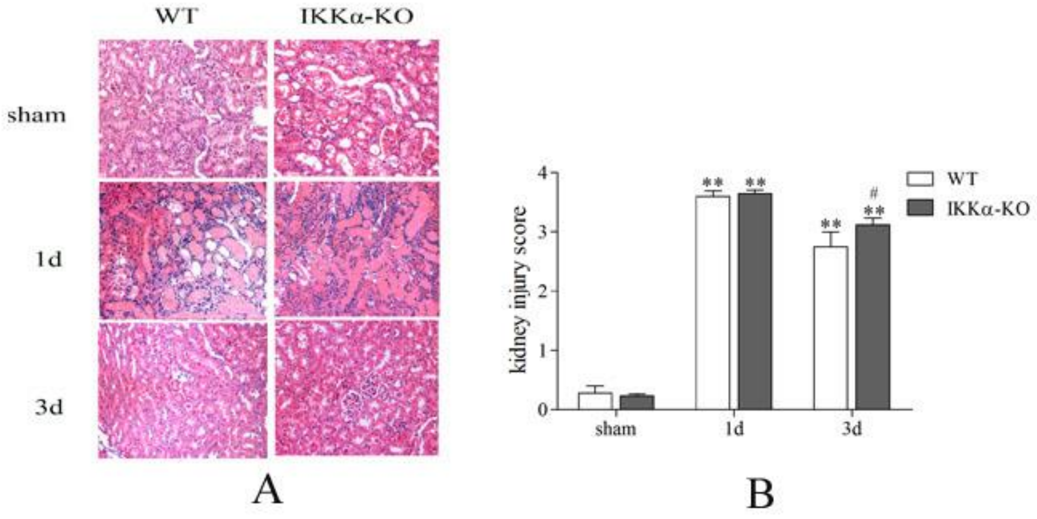

Suppl Fig 2. Renal histological changes on days 1 and 3 following IR injury.

C57BL/6 (WT) or IKKα$^{-/-}$(IKKα-KO) mice underwent sham operation (sham), or conducted IR by unilateral renal pedicle clamping for 45 min, followed by reperfusion. The kidney tissues were harvested on days 1 and 3, respectively.

H&E staining of renal cortex (A) There was severe tubular damage in the kidney of the IKKα-KO mice compared with that of the WT mice. Histological damage score was determined by a skilled staff in the field in a single-blind manner (B).

Data are presented as mean ± SD (n = 6 each). $^{**}P < 0.01$ vs. sham, $^{\#}P < 0.05$ vs. WT.
